# Efficacy and safety of immune checkpoint inhibitors in patients with non‐small cell lung cancer aged 80 years or older

**DOI:** 10.1002/cnr2.1405

**Published:** 2021-05-02

**Authors:** Zentaro Saito, Kohei Fujita, Misato Okamura, Takanori Ito, Yuki Yamamoto, Osamu Kanai, Masayuki Hashimoto, Koichi Nakatani, Satoru Sawai, Tadashi Mio

**Affiliations:** ^1^ Division of Respiratory Medicine, Center for Respiratory Diseases National Hospital Organization Kyoto Medical Center Kyoto Japan; ^2^ Department of Drug Discovery for Lung Diseases, Graduate School of Medicine Kyoto University Kyoto Japan; ^3^ Division of Thoracic Surgery, Center for Respiratory Diseases National Hospital Organization Kyoto Medical Center Kyoto Japan

**Keywords:** elderly patients, immune checkpoint, immunotherapy, lung cancer

## Abstract

**Background:**

In Japan, over 25% of the population is elderly. As the risk of lung cancer increases with age, the number of elderly patients with lung cancer also increases. Given the challenges of an aging society, it is critical that elderly patients receive safe therapies.

**Aim:**

We assessed the safety and efficacy of immune checkpoint inhibitors (ICIs) in patients with non‐small cell lung cancer (NSCLC) aged ≥80 years.

**Methods:**

We retrospectively reviewed NSCLC patients aged ≥80 years old who received ICIs in the National Hospital Organization Kyoto Medical Center. We collected data on patient characteristics, prior treatments, number of cycles, response, and immune‐related adverse events (irAEs) during ICI monotherapy.

**Results:**

A total of 45 patients were reviewed. The patients' median age was 85 years. Twenty‐one, 17, and 7 patients received nivolumab, pembrolizumab, and atezolizumab, respectively. The disease control rate (partial response [PR] + stable disease [SD]) was 60.0%, and the progression‐free survival was 3.4 months. In patients with nivolumab, seven patients (33.3%) achieved SD, and three patients (14.2%) achieved PR. In patients treated with pembrolizumab, seven patients (41.2%) achieved SD, and six patients (35.3%) achieved PR. In patients with atezolizumab, three patients (42.9%) achieved SD, and one patient (14.2%) achieved PR. Sixteen (36%) patients presented with a poor performance status. Three patients treated with pembrolizumab experienced grade 3 pneumonia, while one patient treated with nivolumab experienced grade 5 pneumonia.

**Conclusion:**

This study suggested that ICIs are an acceptable treatment option for NSCLC patients aged ≥80 years. Oncologists should pay attention to severe irAEs.

## 1 INTRODUCTION

Recently, immune checkpoint inhibitors (ICIs) have proven efficacious for the treatment of lung cancer.^1^ In Japan, currently, the anti‐programmed cell death (PD)‐1 antibodies nivolumab and pembrolizumab, and the PD‐ligand 1 (PD‐L1) antibodies atezolizumab and durvalumab have been approved for the treatment of lung cancer. However, durvalumab is only approved for use after chemoradiotherapy, while the other three ICIs can be used for conventional lung cancer treatment.^2^

In Japan, over 25% of the population is elderly, and the number of adults aged 80 years or older has been gradually increasing, emblematic of a super‐aging society. Inevitably, the incidence of lung cancer is higher in older populations, and the median age for the diagnosis of lung cancer is around 70 years.^3^ Given the concerns on the potential for adverse events, it is often difficult to perform conventional cytotoxic chemotherapy in elderly patients. However, a recent report demonstrated that ICIs improved survival in both younger (<65 years old) and older groups (≥65 years old),^4^ indicating that ICIs may represent a better treatment option for elderly patients. However, to date, there have been no reports evaluating the efficacy and safety of ICIs in patients aged 80 years or older.

In this study, we aimed to evaluate the efficacy and safety of ICIs in patients aged 80 years or older with non‐small cell lung cancer (NSCLC).

## 2 PATIENTS AND METHODS

This retrospective cohort study was conducted at the National Hospital Organization Kyoto Medical Center (600 beds), in Kyoto, Japan. We reviewed the clinical data of patients with NSCLC aged 80 years or older who received anti‐PD‐1 antibodies and anti‐PD‐L1 antibodies between December 2015 and April 2020. All patients had pathologically confirmed NSCLC. We included patients treated with nivolumab, pembrolizumab, or atezolizumab ICI monotherapy irrespective of any history of previous cytotoxic, epidermal growth factor receptor (EGFR), or tyrosine kinase inhibitor (TKI) therapy. We administered nivolumab, pembrolizumab, and atezolizumab at 3 mg/kg or 240 mg/body biweekly, 200 mg/body every 3 weeks, and 1200 mg/body every 3 weeks, respectively.

We collected data on patient characteristics, the number of treatment cycles, progression‐free survival (PFS), treatment regimens, best response, and immune‐related adverse events (irAEs). We evaluated the PD‐L1 expression with tumor proportion score (TPS). We divided TPS into four groups as follows: ≥50%, 1%‐49%, <1%, and unknown. Treatment response was evaluated based on the Response Evaluation Criteria in Solid Tumor version 1.1.^5^ Moreover, irAEs were evaluated based on the Common Terminology Criteria for Adverse Events version 5.0.^6^ This study protocol was approved by the Ethical Committee and the Institutional Review Board of the National Hospital Organization Kyoto Medical Center (approval number: 20‐031).

## 3 RESULTS

### 3.1 Patient's characteristics

We reviewed 45 patients with NSCLC who were ≥80 years of age. Patient characteristics are described in Table 1. The median age at initial ICI treatment was 85 years. Patients were more frequently female and had a history of smoking. Regarding comorbidities, 18 (40%), 5 (11%), 20(44%), 9 (20%), and 3 (7%) patients had hypertension, diabetes mellitus (DM), chronic obstructive pulmonary disease (COPD), interstitial pneumonia, and autoimmune disease, respectively. More than two‐thirds of patients were diagnosed with advanced‐stage NSCLC. Twenty‐one patients had adenocarcinoma, of which four patients harbored EGFR mutations. Of the 45 patients, 21 (47%), 17 (38%), and 7 (15%) patients received nivolumab, pembrolizumab, and atezolizumab, respectively. Sixteen (36%) patients presented with poor performance status.

**TABLE 1:** Clinical characteristics of included patients

Characteristics	*n* = 45
Age, years (range)	85 (80‐94)
Gender, Male/Female	10/35
Smoking status	
Current	11 (24)
Past	28 (62)
Never	6 (14)
Comorbidities	
Hypertension	18 (40)
Diabetes mellitus	5 (11)
COPD	20 (44)
Interstitial pneumonia	9 (20)
Autoimmune disease	3 (7)
Clinical stage	
1	6 (13)
2	4 (9)
3	17 (38)
4	18 (40)
Histopathology	
Squamous cell carcinoma	20 (44)
Adenocarcinoma	21 (47)
Not otherwise specified	4 (9)
Driver oncogene alteration	
EGFR mutation	4 (9)
Performance status	
2≤	16 (36)
Immune checkpoint inhibitor	
Nivolumab	21 (47)
Pembrolizumab	17 (38)
Atezolizumab	7 (15)
Number of prior treatments	
1≤	38 (84)

*Note*: Data are expressed as number (%) or median (range).

Abbreviations: COPD, chronic obstructive pulmonary disease; EGFR, epidermal growth factor receptor.

### 3.2 Treatment profiles of ICIs monotherapy in elderly people aged 80 years or older

Table 2 shows the ICI monotherapy treatment profiles used by patients in this study. While seven (15%) patients received pembrolizumab as a first‐line treatment, no patients received nivolumab and atezolizumab as a first‐line treatment. The median number of ICI cycles was four (range, 1‐49). Thirty patients (66.7%) had previously received cytotoxic chemotherapy, of which 2 (4.4%), 2 (4.4%), and 1 (2.2%) patient receiving nivolumab, pembrolizumab, and atezolizumab, respectively, had a history of chemoradiotherapy. No patients had palliative and adjuvant therapy. The disease control rate (PR + SD) was 60.0%. The median PFS was 3.4 months. While patients receiving pembrolizumab had high PD‐L1 expression, patients receiving atezolizumab had low PD‐L1 expression. In the majority of patients who received nivolumab, PD‐L1 expression was not evaluated. The median PFS was longer in patients receiving pembrolizumab (4.6 months) and atezolizumab (5.0 months) than in patients receiving nivolumab (2.3 months). In evaluating the best response, patients receiving pembrolizumab had the highest disease control rate.

**TABLE 2:** Treatment profiles of ICI monotherapies in patients aged 80 or over

	Total	Nivolumab	Pembrolizumab	Atezolizumab
	*N* = 45	*n* = 21 (46.7%)	*n* = 17 (37.8%)	*n* = 7 (15.6%)
Regimens before ICI	2 (0–4)	1 (1–4)	0 (0–3)	1 (1–3)
First‐line treatment	7 (15.6)	0 (0.0)	7 (41.2)	0 (0.0)
Cycles of ICI	4 (1‐49)	3 (1‐49)	4 (1–33)	4 (2‐25)
PD‐L1 expression				
TPS≥50%	14 (31.1)	2 (9.5)	12 (70.6)	0 (0.0)
1% ≤ TPS < 50%	9 (20.0)	1 (4.8)	5 (29.4)	3 (42.9)
TPS <1%	4 (8.9)	2 (9.5)	0 (0.0)	2 (28.6)
unknown	18(40.0)	16 (76.2)	0 (0.0)	2 (28.6)
PFS, month	3.4 (0.2‐17.8)	2.3 (0.2‐3.7)	4.6 (0.5‐13.0)	5.0 (1.0‐17.8)
Best response				
PR	10 (22.2)	3 (14.3)	6 (35.3)	1 (14.2)
SD	17 (37.8)	7 (33.3)	7 (41.2)	3 (42.9)
PD	18 (40.0)	11 (52.4)	4 (23.5)	3 (42.9)
Treatment prior to ICI				
Cytotoxic chemotherapy	30 (66.7)	20 (95.3)	4 (23.5)	6 (85.7)
Radiotherapy	11 (24.4)	4 (19.0)	5 (29.4)	2 (28.6)
Chemoradiotherapy	5 (11.1)	2 (9.5)	2 (11.8)	1 (14.3)
Surgery	3 (6.7)	1 (4.8)	2 (11.8)	0 (0.0)

*Note*: Data are shown as number (%) or median (range).

Abbreviations: PD, progressive disease; PD‐L1, programmed cell death ligand‐1; PFS, progression‐free survival; PR, partial response; SD, stable disease; TPS, tumor proportion score.

### 3.3 Profiles of irAEs

Table 3 shows the profiles of the irAEs. Fatigue and infection were most frequently observed. One patient showed grade 5 pneumonia during nivolumab treatment. Three patients experienced grade 3 pneumonia during pembrolizumab treatment. Among patients with pulmonary toxicity, none of the patients had interstitial pneumonia, autoimmune disease, and radiotherapy previously. Patients receiving atezolizumab tended to have fewer irAEs than other ICIs. No patient treated with atezolizumab experienced a severe irAE. Each individual (2.2%) patient with interstitial pneumonia, anorexia, and diarrhea was treated with corticosteroid to control irAEs. Methylprednisolone (1000 mg/day) was administered to the patient who experienced grade 2 interstitial pneumonia for 3 days.

**TABLE 3:** Profiles of adverse events

	Total		Nivolumab		Pembrolizumab		Atezolizumab	
	G1	G2≤	G1	G2≤	G1	G2≤	G1	G2≤
Fatigue	4	0	1	0	2	0	1	0
Interstitial pneumonia	1	1	1	0	0	1	0	0
Rash	2	0	1	0	1	0	0	0
Pneumonia	0	4	0	1^a^	0	3^b^	0	0
Diarrhea	1	0	0	0	1	0	0	0
Anorexia	1	1	0	1	1	0	0	0
Hoarseness	1	0	0	0	1	0	0	0
Elevation of liver enzyme	1	0	0	0	1	0	0	0
Duodenal perforation	0	1	0	0	0	0	0	1

*Note*: G, grade according to the CTCAE ver 5.0.

^a^ G5 pneumonia.

^b^ G3 pneumonia.

## 4 DISCUSSION

Japan possesses the world's oldest population, and consequently, a major challenge lies in how to manage the high rates of malignant diseases that invariably develop in elderly people. Indeed, the cumulative lifetime risk of cancer incidence, estimated based on cancer incidence data in 2012, is 63% for men and 47% for women.^7^, ^8^ Given its improved tolerability, the adoption of ICIs represents a new innovation for the treatment of elderly patients with cancer. In CheckMate 017, nivolumab reduced the risk of death by 49% in the 65‐75‐years‐old age group; however, no significant hazard ratio for survival was observed in patients aged ≥75.^9^ In Keynote 010, pembrolizumab reduced the risk of death by 37% in patients younger than 65 years. It further reduced the risk of death by 24% in patients aged 65‐69 years.^10^ The OAK trial indicated that atezolizumab improved the survival rate of elderly patients aged ≤75.^11^ A previous study showed that the clinical efficacy and safety of ICIs were not significantly different between cancer patients aged under and over 70 years.^12^ In this study, we showed the efficacy and safety of ICI therapy in very elderly patients. To our knowledge, this study is the first to evaluate the efficacy and safety of ICIs in elderly patients aged 80‐years or older.

A previous study reported that patients aged <75 years treated with ICI showed favorable overall survival and PFS compared with similarly aged patients in the non‐ICI groups.^13^ Moreover, one study showed that in patients with melanoma, anti‐PD‐1 and anti‐PD‐L1 inhibitors resulted in similar overall survival and PFS regardless of age.^14^ Our study showed that ICIs had favorable efficacy and acceptable safety even in patients aged 80 years or older. In particular, patients receiving pembrolizumab achieved a better response (disease control rate: 76.5%) than other ICIs. Because the KEYNOTE 024 trial was built on the success of pembrolizumab monotherapy in patients with NSCLC who have high (50%≤) PD‐L1 expression,^15^ patients with high PD‐L1 expression tended to receive pembrolizumab in our study. This could be one of the reasons why patients receiving pembrolizumab showed a better response than patients receiving other ICIs in our analysis.

In patients receiving nivolumab, 5 (23.8%) patients presented with an irAE of any grade, which is less than the frequency reported in a previous study where 50% of patients sustained an irAE.^16^ However, severe irAEs of grade 5 pneumonia were observed in patients receiving nivolumab. Furthermore, severe irAEs of grade 3 pneumonia were also observed in patients receiving pembrolizumab. Anti‐PD‐1 antibodies seemed to cause more severe irAEs than anti‐PD‐L1 antibodies in our study. Consequently, it is prudent to give greater attention to the development of severe irAEs when treating elderly patients, especially those treated with anti‐PD‐1 antibodies. Judging from the profiles of irAEs in our study, we propose that atezolizumab is a safer choice than the other ICIs that were tested. Although our study showed several severe irAEs, the type and grade of general adverse events related to ICIs were largely similar to those of a previous study.^17^ Severe treatment‐related adverse events occurred less frequently with nivolumab and pembrolizumab than with docetaxel.^18^ In addition, atezolizumab demonstrated a favorable safety profile compared with docetaxel.^19^ Therefore, it seems that in elderly people with poor performance status (PS), ICI monotherapy can be a more suitable option than conventional cytotoxic chemotherapy.

There were several limitations to our study. First, this study was retrospective in nature and was conducted in a single institution. Moreover, we only included a small sample size, and invariably suffered from bias in the selection of participants. Second, although all patients received ICIs, we did not consider the effects of regimens prior to ICIs. Thirty‐eight (84%) patients had at least 1 cycle of cytotoxic chemotherapy and a molecular target drug prior to ICIs. This may have induced a depressive immune response. Consequently, we cannot rule out that using ICIs as a first‐line regime would produce better results. Third, the timing of treatment was chosen by the attending doctors; therefore, it was not standardized between patients.

In conclusion, ICI monotherapy, especially pembrolizumab and atezolizumab, is a reasonable treatment option for patients with lung cancer aged 80 years or older. More attention in the clinic should be placed on evaluating the development of severe irAEs during immunotherapy.

## CONFLICT OF INTEREST

The authors have stated explicitly that there are no conflicts of interest in connection with this article.

## AUTHOR CONTRIBUTIONS

*Conceptualization; data curation; formal analysis; investigation; methodology; writing‐original draft; writing‐review & editing*, Z.S.; *Conceptualization; data curation; formal analysis; funding acquisition; investigation; methodology; project administration; resources; software; supervision; validation; visualization; writing‐original draft; writing‐review & editing*, K.F.; *Data curation; project administration; supervision*, M.O.; *Methodology; project administration; supervision*, T.I. and Y.Y.; *Investigation; project administration; supervision; validation*, O.K.; *Investigation; project administration; supervision*, M.H.; *Investigation; methodology; supervision*, K.N. and S.S.; *Conceptualization; investigation; methodology; supervision*, T.M.

## ETHICS STATEMENT

This study protocol was approved by the Ethical Committee and the Institutional Review Board of the National Hospital Organization Kyoto Medical Center (approval number: 20‐031).

## Data Availability

All data are available upon reasonable request after IRB approval.
